# Infralimbic cortex controls fear memory generalization and susceptibility to extinction during consolidation

**DOI:** 10.1038/s41598-020-72856-0

**Published:** 2020-09-28

**Authors:** Hugo Bayer, Leandro Jose Bertoglio

**Affiliations:** Departamento de Farmacologia, CCB, Universidade Federal de Santa Catarina (UFSC), Campus Universitário s/n, Florianópolis, SC 88049-900 Brazil

**Keywords:** Consolidation, Fear conditioning

## Abstract

Lesioning or inactivating the infralimbic (IL) subregion of the medial prefrontal cortex before acquisition produces more generalized and extinction-resistant fear memories. However, whether and how it modulates memory specificity and extinction susceptibility while consolidation takes place is still unknown. The present study aims to investigate these questions using muscimol-induced temporary inactivation and anisomycin-induced protein synthesis inhibition in the rat IL following contextual fear conditioning. Results indicate that the IL activity immediately after acquisition, but not six hours later, controls memory generalization over a week, regardless of its strength. Such IL function depends on the context-shock pairing since muscimol induced no changes in animals exposed to immediate shocks or the conditioning context only. Animals in which the IL was inactivated during consolidation extinguished similarly to controls within the session but were unable to recall the extinction memory the following day. Noteworthy, these post-acquisition IL inactivation-induced effects were not associated with changes in anxiety, as assessed in the elevated plus-maze test. Anisomycin results indicate that the IL protein synthesis during consolidation contributes more to producing extinction-sensitive fear memories than memory specificity. Collectively, present results provide evidence for the IL's role in controlling generalization and susceptibility to extinction during fear memory consolidation.

## Introduction

To cope with life-threatening events, most animals rely on aversive memories and, thus, the appropriate formation of them are essential and conserved across species^1,2^. Aversive memories may vary according to quantitative (e.g., intensity) and qualitative (e.g., specificity) aspects^3^. The study of when, how, and where into the brain such features are established is scientifically relevant, and contributes to discovering/developing better interventions that could have therapeutic value to tackle traumatic memories^4,5^, which are commonly overgeneralized and more resistant to extinction^6,7^.

Newly acquired aversive memories require a gradual consolidation to become stable and long-lasting^8^. This process involves the activity and plasticity in several interconnected brain regions, including the hippocampus, amygdala, and medial prefrontal cortex^9^. Interventions aiming at temporarily inactivating their activity or inhibiting their consolidation-associated protein synthesis carried out immediately after acquisition have been shown by many to affect memory intensity^10^. On the other hand, how specific (or generalized) and prone to extinction is the aversive memory when these experimental approaches are performed during consolidation have been less addressed.

The rodent infralimbic (IL) subregion of the medial prefrontal cortex has been shown to modulate the specificity and generalization attributes of cued and contextual fear memories at the acquisition stage: pre-training damage to the IL^11^ or inactivation of both IL and prelimbic (PL)^12^ was sufficient to produce memory overgeneralization. Similarly, lesioning or inactivating the IL before cued or contextual fear memory acquisition induced a relative resistance to extinction^11,13,14^. The IL has also been associated with aversive memory consolidation. Zhang et al.^15^ reported an increase in the induction of c-fos and Arc expression in IL during inhibitory avoidance memory consolidation. Torres-García et al.^16^ reported that temporary IL inactivation with tetrodotoxin impaired the consolidation of inhibitory avoidance training. In contrast, there were no effects of temporary IL inactivation with muscimol on inhibitory avoidance memory consolidation^17^. Studies focusing on cued or contextual fear memory consolidation have also reported changes in the IL, including depressed intrinsic neuronal excitability^18,19^ and increased Arc expression^20^, but not c-fos induction or histone acetylation^21,22^. Post-acquisition gamma irradiation potentiated the consolidation of a contextual fear memory trace, an effect associated with reduced IL neuronal activation^23^. Together, these findings suggest that associative learning of aversive events induces complex patterns of IL activation and plasticity. However, whether IL influences memory specificity/generalization and extinction susceptibility while consolidation occurs is yet to be investigated.

Based on the above, the present study sought to investigate the effects of muscimol-induced temporary inactivation and anisomycin-induced protein synthesis inhibition in the rat IL during the consolidation of contextual fear memory on its intensity, specificity, and subsequent extinction. The working hypothesis was that the memory would become more intense, generalized, and less prone to extinction following these interventions. Since either lesioning or inactivating the rat IL has occasionally been reported to interfere with anxiety^24,25^, we investigated whether anxiety-related changes could contribute to post-acquisition IL inactivation effects on contextual fear memory intensity, generalization, and susceptibility to extinction. To this aim, animals were tested in the elevated plus-maze shortly after contextual fear conditioning and IL infusion of muscimol or vehicle.

## Materials and methods

### Animals

A total of 196 male Wistar rats aged 13–15 weeks was used in the present study. Animals were obtained from local breeding facilities, kept in groups of 3–4 in polypropylene cages (50 × 30 × 15 cm) on a 12 h light/dark cycle, with lights on at 7 AM, and had free access to water and standard laboratory chow*.* The Institutional Ethical Committee for the Care and Use of Laboratory Animals from our University approved this study in compliance with Brazilian legislation and the National Institutes of Health guide for the care and use of laboratory animals (NIH Publications No. 8023, revised 1978).

### IL stereotaxic surgery

Animals were deeply anesthetized using 1.0 ml/kg of a solution of xylazine (10 mg/ml; Syntec, Brazil) and ketamine (100 mg/ml; Syntec, Brazil), placed in a stereotaxic frame, then local anesthesia with lidocaine 3.0% and epinephrine 1:50,000 (Dentsply, Brazil) was applied. Two stainless steel guide cannulae (22 G, 11 mm) were implanted aimed at the IL cortex following coordinates (AP: + 3.2 mm from Bregma, ML: + 0.6 mm, DV: − 2.1 mm) from the rat brain atlas by Paxinos and Watson^26^, and fixed to the skull using acrylic resin and two stainless steel screws. An obturator (30G) was inserted inside each guide cannula to reduce possible occlusion. For post-surgery analgesia, anti-inflammatory, and antipyretic actions, animals received an intramuscular injection of flunixin meglumine (2.5 mg/kg; Schering-Plough, Brazil). Animals were given at least ten days of recovery before behavioral testing.

### Drugs and infusion procedure

The GABA_A_ receptor agonist muscimol (MUS; Tocris, USA), which suppresses the neurophysiologic activity within 0.5–1.0 mm of the infusion site^27^, was brought to a final concentration of 0.46 μg/μl using phosphate-buffered saline (PBS) 0.1 M. The protein synthesis inhibitor anisomycin (ANI; Sigma-Aldrich, USA), which inhibits most of de novo protein synthesis for at least 3 h^28^, was dissolved in PBS 0.1 M to obtain a final concentration of 100 μg/μl. PBS 0.1 M served as the vehicle for both drugs.

On the day of intra-IL infusion, obturators were removed, and the treatment was delivered using two 14 mm dental needles (30G) connected to microsyringes of 5.0 μl by polyethylene tubing (PE10) inserted into the guide cannulae while the animal was gently restrained with a towel. Either 0.2 μl of drug or vehicle solution was given bilaterally for one min using an infusion pump. The needles remained inside guide-cannulae for one min after the end of injections to reduce possible drug backflow.

### Contextual fear conditioning and elevated plus-maze apparatuses, behavioral procedures, and data collection

Most aspects mentioned in this section were conducted as fully described elsewhere^20,29,30^. Briefly, contextual fear conditioning was performed in a rectangular chamber (35 × 20 × 30 cm), with aluminum sidewalls and a front wall and ceiling-door made of Plexiglas, designated herein as context A. Its grid floor, made of stainless steel bars, was connected to a circuit board and a shock generator (Insight, Brazil) to enable the delivery of controlled electrical shocks, as subsequently detailed. The elevated plus-maze was made of wood and consisted of two opposite open-arms (50 × 10 cm) surrounded by a 1.0 cm high Plexiglas edge and two enclosed-arms (50 × 10 × 40 cm), set up 50 cm above the floor. The junction area of the four arms (central platform) measured 10 × 10 cm.

Behavioral procedures were conducted in rooms under the illumination of 70 lx, from 9 AM to 5 PM. Unless otherwise specified (experiment 6), on day 1 each animal was placed alone in context A and allowed to explore it freely for 3 min, as a familiarization session, and then returned to its home cage.

On day 2, each animal was again placed in context A for fear conditioning, during which it received, after an initial 30 s delay, the unconditioned stimulus (US), which consisted of 3 electrical shocks of 1.0 mA for 3 s, with a 30 s inter-trial interval. The animal remained in this chamber for another 30 s before returning to its home cage. The treatment was given immediately after this session, except in experiment 2, where it was given six hours later as a control since most interventions no longer influence memory consolidation when given at this time-point^8^.

In experiment 6, to investigate whether drug-induced effects depend on the shock-context pairing, the day 2 procedure also included either animals merely exposed to the context A (the CTX only group) or animals not familiarized with context A in which three electrical shocks (1.0 mA for 3 s, with a 5 s inter-trial interval) were delivered in a 20 s pseudo-conditioning session (the US only group). Of note, neither fear conditioning^31^ nor learning-related changes in the IL excitability^19^ were induced by similar pseudo-conditioning procedures.

To assess the treatment effects on memory intensity and specificity, in the following days, animals were exposed for 3 min to the conditioned context A (Test A) and the novel, unpaired context B (Test B), which consisted of a square chamber (30 × 30 × 30 cm) with glass sidewalls and floor, and a white grid lid. In experiment 4, Tests A and B were performed seven and eight days after contextual fear conditioning, respectively.

To assess the treatment effects on memory extinction, in experiments 7 and 9, animals underwent a 15-min session (further divided into five blocks of 3 min) of exposure to the conditioned context A without US presentation one day after contextual fear conditioning. Tests A and B were performed 6 and 7 days later, respectively, to evaluate extinction learning effectiveness.

The time spent freezing, defined as the ceasing of all body and head movements, except the flank movements related to breathing^32^, was used as a fear memory index for all experiments. Such response was quantified in seconds by a trained observer (inter- and intra-observer reliabilities ≥ 90%) blind to the experimental groups and expressed as the percentage of total session time.

A five min test in the elevated plus-maze was conducted as previously described^33^. A trained observer blind to the experimental design scored the following behavioral measures from the DVD: the number of open- and enclosed-arms entries (EAE) with the four paws as well as the time spent in open- and enclosed-arms. Raw data were used to calculate the percentage of entries {%OAE; [open-arms entries/(open- + enclosed-arms entries)] × 100} and time spent in open-arms {%OAT; [(time in open-arms/300) × 100]}. The number of stretched-attend postures (SAPs), defined as a posture in which the subject stretches forward and then retracts to its original position, performed from the central platform or enclosed-arms towards open-arms, was also recorded. It has been shown that the animal's anxiety response level may be inferred from inhibitory avoidance (%OAT and %OAE) and risk assessment (SAPs) behaviors, and the number of EAE is considered an elevated plus-maze index of general exploratory activity^34^.

### Histology

**Figure 1:**
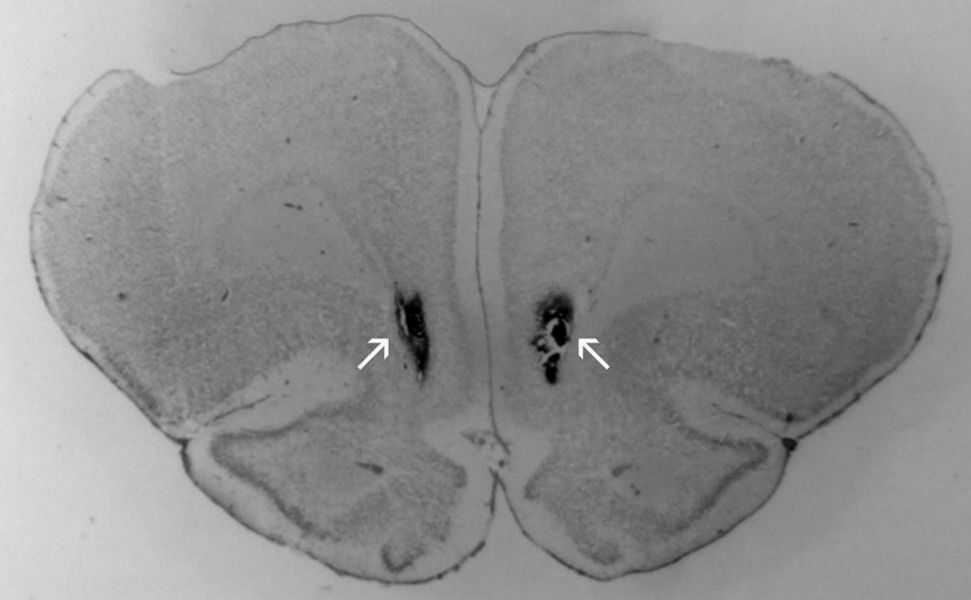
Photomicrograph of representative infusion sites placement (indicated by arrows) in the rat infralimbic cortex (~ 3.2 mm anterior to Bregma).

Most aspects mentioned in this section were conducted as fully described elsewhere^20,29,30^. Briefly, at the end of the experiments, animals were anesthetized as already described for surgery, received a lethal dose of chloral hydrate (3.0 ml/kg of a 15% solution). Then Evans Blue (0.2 μl) was infused into the IL with the same needle used in the respective experiment for subsequent evaluation of the infusion site. Brains were removed and immersed in a 4.0% formalin solution and, later, sucrose 30%, for 48 h each. Then, brain slices (50 µm thick) were obtained in a cryostat, mounted on glass microscope slides, and stained with cresyl violet to determine the infusion site. Its location in the IL ranged from 3.7 to 2.7 mm anterior to Bregma. Figure 1 shows a photomicrograph of the rat IL with infusion sites. Animals receiving the treatment outside this medial prefrontal cortex subregion (about 20%) were excluded from the analysis.

### Statistical analysis

After ensuring the assumptions of normality with Shapiro–Wilk’s W test and homogeneity of variance with Levene’s test, freezing times expressed during Tests A and B were subjected to a mixed analysis of variance (ANOVA) in which the independent factors were treatment and test sessions. The same approach was used to compare the time spent freezing in the first extinction session block with that expressed during Test A. For extinction session data, a mixed ANOVA was carried out in which the independent factors were treatment and time-bin. The Newman–Keuls test was used for *post-hoc* multiple comparisons in the abovementioned cases. The discrimination index (the difference between the percentage of time spent freezing during Test A and Test B, divided by the sum of the two percentages), the elevated plus-maze data, and the delta of the freezing time (the difference between the percentage of time spent freezing during the first extinction block and Test A) used in experiments 7 and 9 were analyzed using two-sample unpaired Student t-tests. The statistical significance level was set at *p* < 0.05. For statistical analysis, Statistic 13.5 (StatSoft, EUA) was used, and GraphPad Prism 8.02 (GraphPad Prism, EUA) was used for graphing.

The effect size was calculated using the formula for Hedges’ *g* to reflect the mean-difference between two groups (n ≤ 20 per group) that could be dissimilar in size. A *g* ≥ 0.8 was considered a large effect size^35^. The sample size determined by power analysis was 8 animals per group (α = 0.05; β = 0.20 and standardized effect size or Cohen's *d* = 1.0). The group sizes were equal by design, but due to experimental losses (when treatment was infused outside the target brain region) or the violation of the predetermined exclusion criterion (fear-conditioned animals were those spending at least 35% of freezing behavior during Test A or first extinction block, except for the experiment 4 where there was a single context-shock pairing), in a few cases, they were unequal. We have replaced the exclusions in an attempt to keep the study balanced and to maintain its power.

## Results

### Experiment 1: Effects of IL inactivation during the consolidation of a contextual fear memory on its intensity and specificity

**Figure 2:**
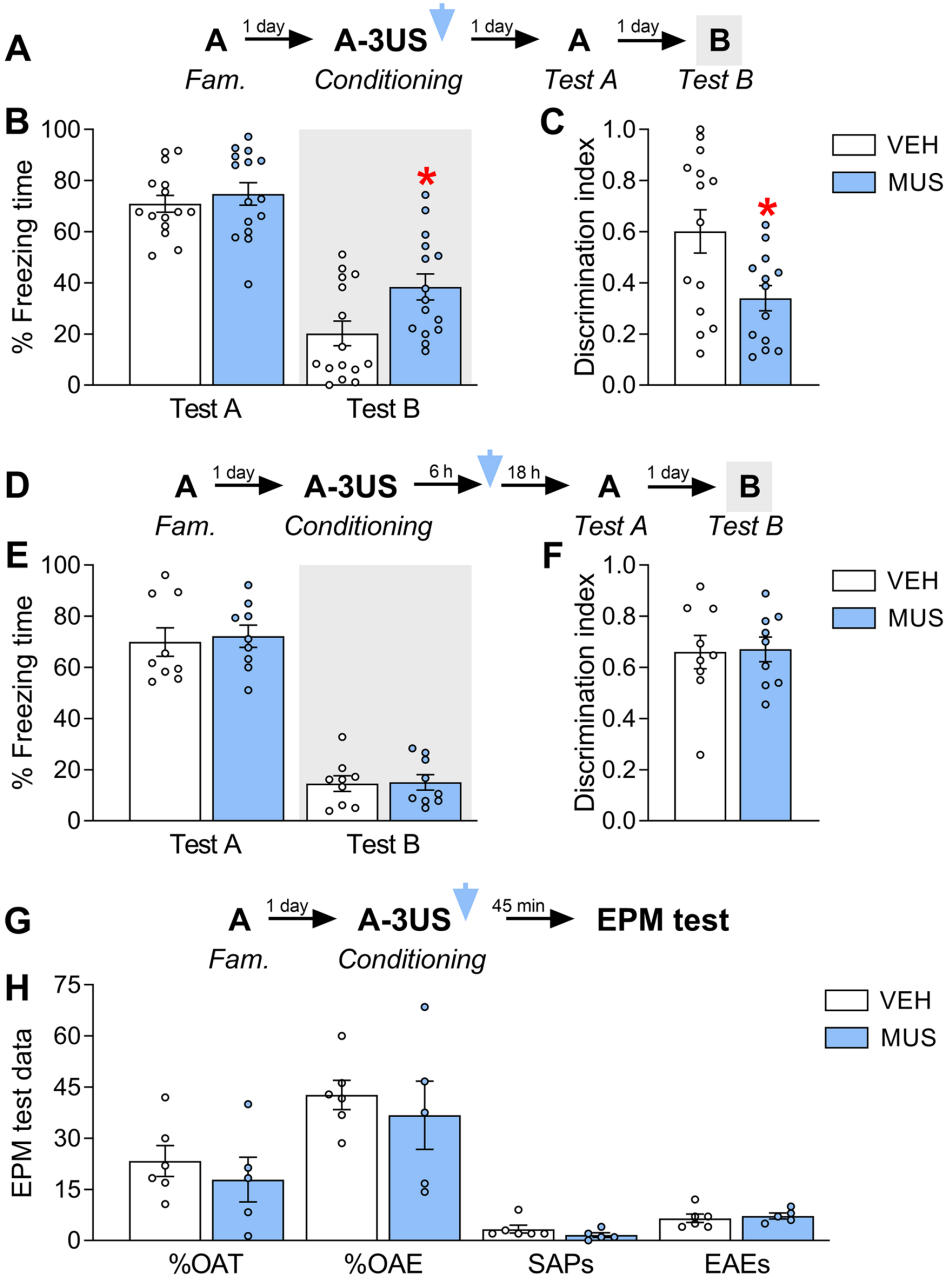
Effects of IL inactivation by muscimol (MUS) immediately (*upper graphs*) or six hours after (*middle graphs*) the acquisition of contextual fear memories on their expression in the paired context A (Test A) and the novel, neutral context B (Test B), and on anxiety-related behavior and general exploratory activity assessed in the elevated plus-maze (EPM) test 45 min later (*lower graph*). (**A**) Experiment 1′s design. (**B**) MUS-treated animals presented comparable freezing times to controls (VEH) during Test A. However, they had higher levels during Test B. (**C**) The MUS group presented lower discrimination index values than the VEH group. (**D**) Experiment's 2 design. (**E**) MUS-treated animals presented comparable freezing times to VEH during both Tests A and B. (**F**) The MUS group presented similar discrimination index values to the VEH group. (**G**) Experiment's 3 design. (**H**) MUS-treated animals and controls behaved similarly in either case [anxiety: % open-arm time (%OAT), % open-arm entries (%OAE) and stretched-attend postures (SAPs); general exploratory: enclosed-arm entries (%EAEs)]. Values are expressed as individual units and mean ± S.E.M. (*n* per group in experiment 1 = 15; experiment 2 = 9; and experiment 3 = 5–6). The * denotes a statistically significant difference (*p* < 0.05) from the respective control group (mixed ANOVA followed by the Newman–Keuls test or unpaired t-test).

Thirty animals were randomly allocated to two groups based on the treatment (vehicle, n = 15; muscimol, n = 15) given immediately after pairing the context A with three shocks (Fig. 2A). In the following days, both groups were exposed to the conditioned context (Test A) and the novel, neutral context B (Test B) in a counterbalanced order (i.e., half of animals of each group performed Test B before Test A). In both groups, data of the latter animals did not differ qualitatively from those in which Test A was performed before Test B. Therefore, they were merged for the statistical analysis. To assess treatment effects on memory specificity and generalization, data from Tests A and B were used to calculate the discrimination index.

A mixed ANOVA showed significant effects of treatment (F_1,28_ = 4.2; *p* = 0.04), test sessions (F_1,28_ = 166.1; *p* = 0.00001), and their interaction (F_1,28_ = 4.5; *p* = 0.04) for freezing time. As shown in Fig. 2B, Newman–Keuls *post-hoc* tests showed that muscimol and vehicle groups behaved similarly during Test A (*p* = 0.61; Hedges’ *g* effect size = 0.25), but during Test B animals treated with muscimol presented higher levels than controls (*p* = 0.02; *g* = 0.94). Furthermore, there were significant treatment effects for the discrimination index (*t*_1,28_ = 8.1; *p* = 0.008). As shown in Fig. 2C, muscimol-treated animals presented lower values than controls (*p* = 0.008; *g* = 1.04).

### Experiment 2: Effects of IL inactivation six hours after the acquisition of a contextual fear memory on its intensity and specificity

To test whether the abovementioned findings depend on the time elapsed between fear conditioning and IL inactivation, 18 animals had the context A paired with three shocks but only received the treatment (vehicle, n = 9; muscimol, n = 9) six hours later (Fig. 2D). Tests A and B were performed on the following days. Their data were used to calculate the discrimination index.

A mixed ANOVA showed significant effects of test sessions (F_1,16_ = 211.47; *p* = 0.00001), but not treatment (F_1,16_ = 0.10; *p* = 0.75) or interaction between these factors (F_1,16_ = 0.05; *p* = 0.81) for freezing time. As shown in Fig. 2E, muscimol and vehicle groups behaved similarly during both Tests A and B. Regarding the discrimination index, there were also no significant treatment effects (*t*_1,16_ = 0.17; *p* = 0.90) (Fig. 2F).

### Experiment 3: Acute effects of IL inactivation immediately after contextual fear memory acquisition on the expression of anxiety-related behaviors

To investigate the possibility that augmented freezing time seen during Test B performed 24 h after fear conditioning and IL inactivation (Experiment 1) was associated with changes in anxiety-related behaviors, 11 animals had context A paired with three shocks and then were randomly allocated to two groups based on the treatment (vehicle, n = 6; muscimol, n = 5) given immediately after that. Both groups performed the elevated plus-maze test 45 min later (Fig. 2G).

Unpaired Student's t-tests showed no significant effects of treatment for inhibitory avoidance (%OAT: *t*_9_ = 0.70, *p* = 0.50 and %OAE: *t*_9_ = 0.58, *p* = 0.57), risk assessment (SAPs: *t*_9_ = 1.2; *p* = 0.25), and general exploratory activity (EAE: *t*_9_ = 0.31, *p* = 0.76) in the elevated plus-maze test. As shown in Fig. 2H, muscimol-treated rats did not differ significantly from controls in any of these behavioral measures, indicating that post-acquisition IL inactivation effects were not due to altered levels of anxiety during consolidation.

### Experiment 4: Effects of IL inactivation during the consolidation of a weaker contextual fear memory on its intensity and specificity

**Figure 3:**
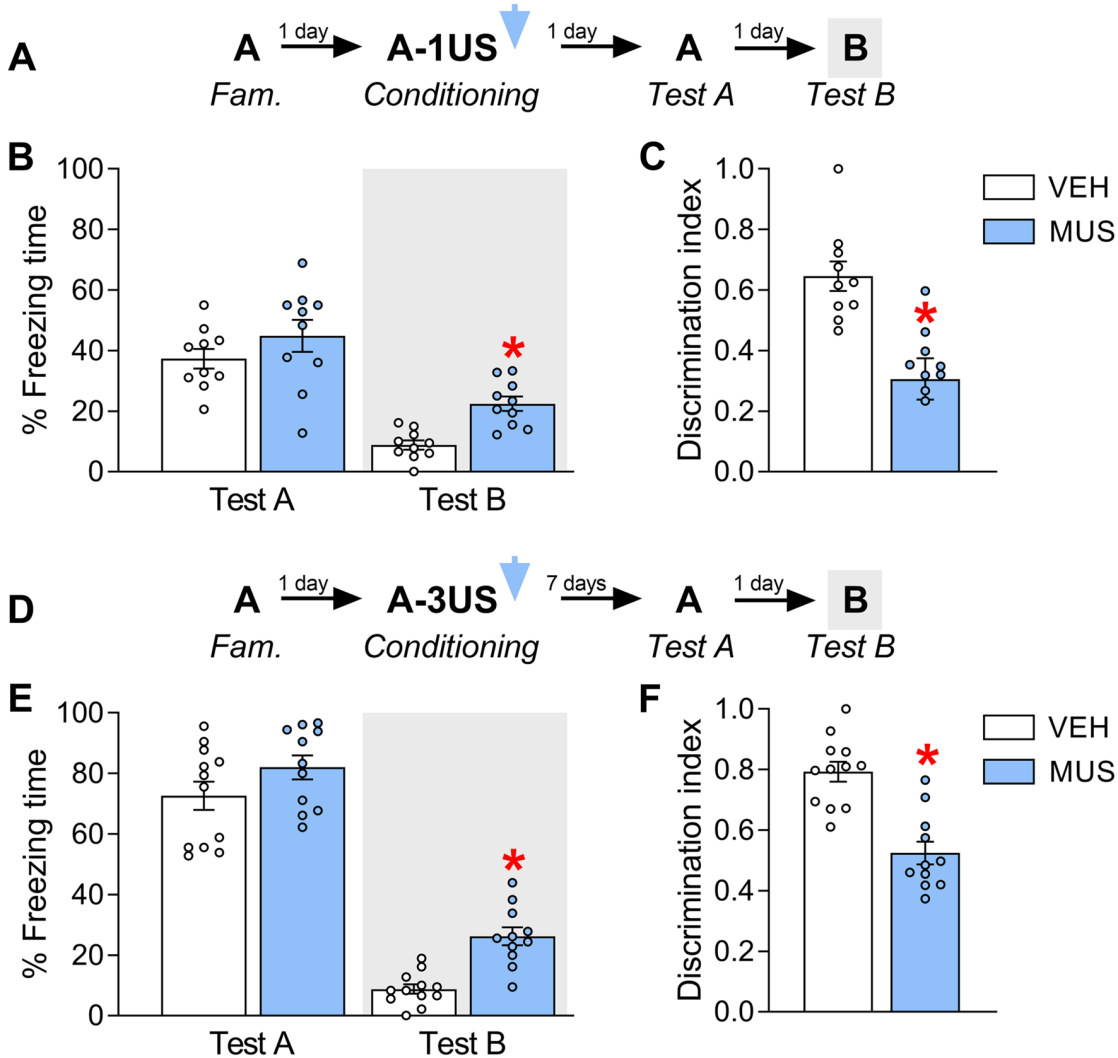
Effects of IL inactivation by muscimol (MUS) immediately after weak contextual fear memory acquisition on expression in the paired context A (Test A) and the novel, neutral context B (Test B) evaluated at a recent time point (*upper graphs*), or after strong contextual fear memory acquisition evaluated at a more remote time point (*lower graphs*). (**A**) Experiment 4′s design. (**B**) MUS-treated animals presented comparable freezing times to controls (VEH) during Test A. However, they had higher levels during Test B. (**C**) The MUS group presented lower discrimination index values than the VEH group. (**D**) Experiment's 5 design. (**E**) MUS-treated animals presented comparable freezing times to VEH during Test A, although they had higher levels during Test B. (**I**) The MUS group presented lower discrimination index values than the VEH group. Values are expressed as individual units and mean ± S.E.M. (*n* per group in experiment 4 = 10; and experiment 5 = 11–12). The * denotes a statistically significant difference (*p* < 0.05) from the respective control group (mixed ANOVA followed by the Newman–Keuls test or unpaired t-test).

Freezing times close to the ceiling level could have concealed possible differences between groups during Test A in experiment 1. To evaluate this possibility, 20 animals were randomly allocated to two groups based on the treatment (vehicle, n = 10; muscimol, n = 10) given immediately after pairing the context A with a single shock (Fig. 3A). Tests A and B were performed on the following days, and their data were used to calculate the discrimination index.

A mixed ANOVA showed significant effects of treatment (F_1,18_ = 6.5; *p* = 0.02), test sessions (F_1,18_ = 106.6; *p* = 0.00001), but not their interaction (F_1,18_ = 1.5; *p* = 0.23), for freezing time. As shown in Fig. 3B, unprotected Newman–Keuls tests showed that muscimol and vehicle groups behaved similarly during Test A (*p* = 0.21; *g* = 0.54), but animals treated with muscimol presented higher levels than controls during Test B (*p* = 0.03; *g* = 2.16). Moreover, there were significant treatment effects for the discrimination index (*t*_1,18_ = 16.3; *p* = 0.001). As shown in Fig. 3C, muscimol-treated animals presented lower values than controls (*p* = 0.001; *g* = 1.80).

### Experiment 5: Effects of IL inactivation during the consolidation of a contextual fear memory on its intensity and specificity evaluated at a more remote time point

To investigate whether IL inactivation-induced effects persist for longer periods, 23 animals were randomly allocated to two groups based on the treatment (vehicle, n = 12; muscimol, n = 11) given immediately after pairing the context A with three foot-shocks (Fig. 3D). Tests A and B were performed 7 and 8 days later.

A mixed ANOVA showed significant effects of treatment (F_1,21_ = 10.9; *p* = 0.003), test sessions (F_1,21_ = 450.2; *p* = 0.00001), but not their interaction (F_1,21_ = 2.1; *p* = 0.17), for freezing time. As shown in Fig. 3E, unprotected Newman–Keuls tests showed that muscimol and vehicle groups behaved similarly during Test A (*p* = 0.12; *g* = 0.63), but animals treated with muscimol presented higher levels than controls during Test B (*p* = 0.006; *g* = 2.21). Moreover, there were significant treatment effects for the discrimination index (*t*_1,21_ = 28.8; *p* = 0.0001). As shown in Fig. 3F, muscimol-treated animals presented lower values than controls (*p* = 0.0001; *g* = 2.23).

### Experiment 6: Are the effects of IL inactivation on fear generalization learning-dependent?

**Figure 4:**
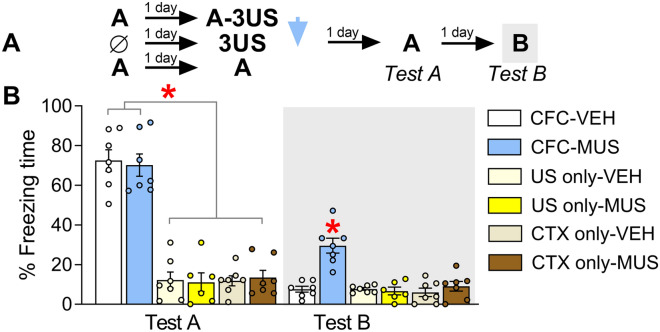
Effects of IL inactivation by muscimol (MUS) after pairing the context A with three shocks (CFC), exposure to immediate foot-shocks (US only) or the mere exposure to the context A (CTX only), on freezing time expressed during exposure to contexts A (Test A) and B (Test B). (Experiment 6). (**A**) The experimental design used. (**B**) During Test A, both CFC-VEH and CFC-MUS groups expressed significantly higher freezing levels than the others did. The CFC-MUS group presented higher freezing levels than the others did during Test B. Values are expressed as individual units and mean ± S.E.M. (n per group = 6–7). The * denotes a statistically significant difference (*p* < 0.05) from all the other groups in the same session (mixed ANOVA followed by the Newman–Keuls test).

To examine this question (i.e., whether muscimol-induced effects are not associated with fear sensitization or drug effects per se), 41 animals were allocated to six groups based on the procedure they underwent on days 1 and 2 (context A-shock pairing—CFC—as in experiment 1; US only or CTX only), and the treatment (vehicle or muscimol) given immediately after that (Fig. 4A). Tests A and B were performed on the following days. The experimental groups were as follow: CFC-VEH, n = 7; CFC-MUS, n = 7; US only-VEH, n = 7; US only-MUS, n = 6; context only-VEH, n = 7; and context only-MUS, n = 7.

A mixed ANOVA showed a significant day 2 procedure *versus* treatment *versus* test sessions interaction (F_2,35_ = 4.8; *p* = 0.01) for freezing time. As shown in Fig. 4B, CFC-MUS and CFC-VEH groups behaved similarly during Test A (*p* = 0.67; *g* = 0.16) but presented higher freezing time levels than the other groups (*p* ≤ 0.0001; *g* ≥ 4.38), which did not differ from each other (*p* ≥ 0.89; *g* ≤ 0.03). During Test B, the CFC-MUS group presented higher levels than the others (*p* ≤ 0.009; *g* ≥ 2.07).

### Experiment 7: Effects of IL inactivation during the consolidation of a contextual fear memory on its subsequent extinction

**Figure 5:**
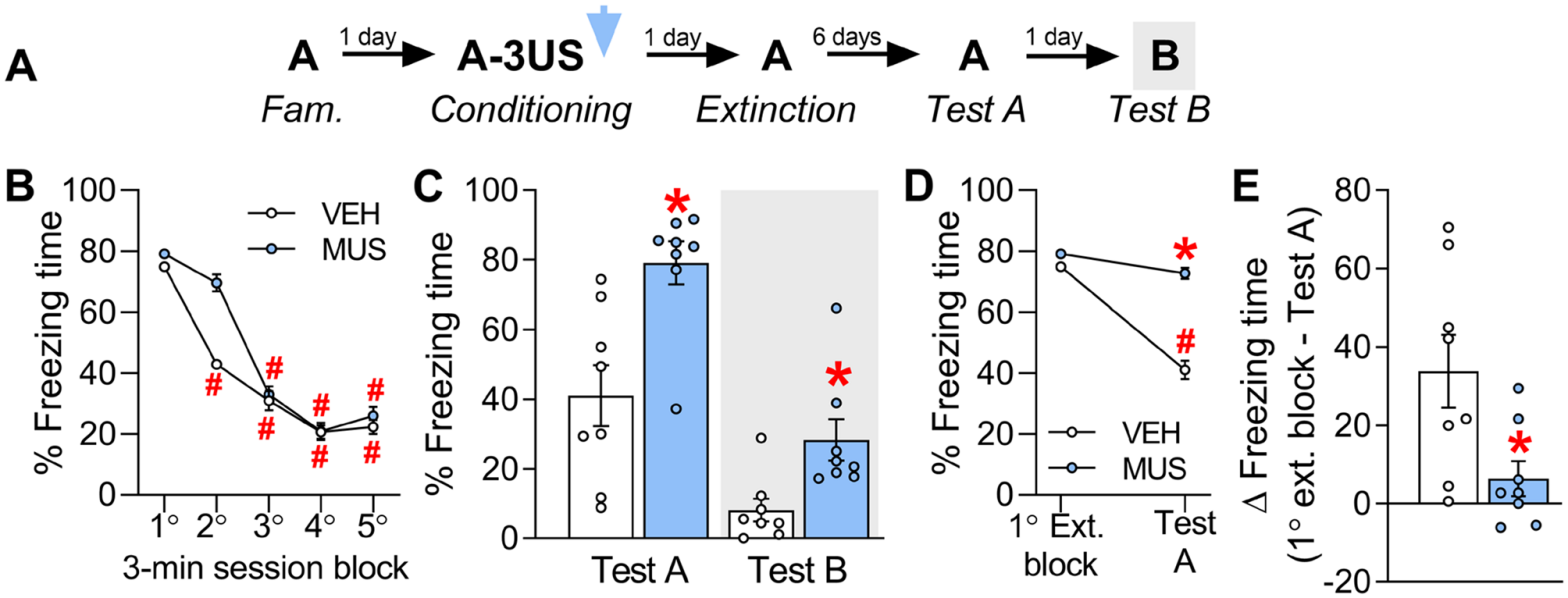
Effects of IL inactivation by muscimol (MUS) during the consolidation of a contextual fear memory on its extinction, and expression in the paired context A (Test A) and the context B (Test B) (Experiment 7). (**A**) The experimental design used. (**B**) The freezing time reduction throughout the extinction session in MUS-treated animals was similar to controls (VEH group). (**C**) MUS-treated animals did not retain the extinction learning as well as the VEH group, as observed during Test A. On Test B, they kept expressing more freezing time than controls. (**D**) To estimate how efficient the extinction learning and memory process was, the time spent freezing in the first extinction session block and that expressed during Test A were compared. There was a difference indicative of a sufficient extinction in animals treated with VEH, but not MUS. As a result, the freezing time delta was lower in MUS-treated animals than controls (**E**). Values are expressed as individual units and/or mean ± S.E.M (n per group = 8). The # represents a significant difference (*p* < 0.05) from the respective first extinction session block (mixed ANOVA followed by the Newman–Keuls test). The * denotes a statistically significant difference from the respective control group (mixed ANOVA followed by the Newman–Keuls test or unpaired t-test).

To determine whether IL inactivation during consolidation interferes with subsequent extinction learning and retention, 16 animals were randomly allocated to two groups based on the treatment (vehicle, n = 8; muscimol, n = 8) given immediately after pairing the context A with three shocks (Fig. 5A). On the next day, both groups underwent a 15-min extinction session (context A re-exposure without US presentation). Test A (to assess the recall of extinction learning) and Test B (to assess whether extinction learning influences freezing expressed in context B) were performed six and seven days later.

For extinction session data, a mixed ANOVA showed significant effects of time-bin (F_4,56_ = 52.7; *p* = 0.00001) and interaction between time-bin and treatment (F_4,56_ = 2.7; *p* = 0.04), but not treatment (F_1,14_ = 1.0; *p* = 0.32). As shown in Fig. 5B, whereas vehicle-treated animals presented lower freezing time values from the second to the fifth extinction session block relative to the first one (*p* ≤ 0.0001; *g* ≥ 4.71), those treated with muscimol only presented a significant reduction from the third to the fifth extinction session block (*p* ≤ 0.0001; *g* ≥ 2.81). No differences between groups were found across any extinction session block (*p* ≥ 0.11; *g* ≤ 1.56).

For Tests A and B data, a mixed ANOVA showed significant effects of test sessions (F_1,14_ = 45.5; *p* = 0.0001) and treatment (F_1,14_ = 15.7; *p* = 0.004), but not their interaction (F_1,14_ = 1.0; *p* = 0.33). As shown in Fig. 5C, unprotected Newman–Keuls tests showed that muscimol-treated animals presented higher freezing time levels than controls during both Test A (*p* = 0.005; *g* = 1.54) and Test B (*p* = 0.05; *g* = 1.49).

To estimate how efficient the extinction learning and memory process was, the time spent freezing in the first 3-min extinction session block (Fig. 5B) and that expressed during the 3-min Test A (Fig. 5C) were compared. A mixed ANOVA showed significant effects of treatment (F_1,14_ = 10.2; *p* = 0.01), test sessions (F_1,14_ = 15.0; *p* = 0.02), and their interaction (F_1,14_ = 7.0; *p* = 0.02). As shown in Fig. 5D, there were reduced values in animals treated with vehicle (*p* = 0.001; *g* = 1.89), but not muscimol (*p* = 0.67; *g* = 0.60). Similarly, as shown in Fig. 5E, there were significant treatment effects for the freezing time delta (*t*_1,14_ = 6.9; *p* = 0.02).

### Experiment 8: Effects of IL protein synthesis inhibition during the consolidation of a contextual fear memory consolidation on its intensity and specificity

**Figure 6:**
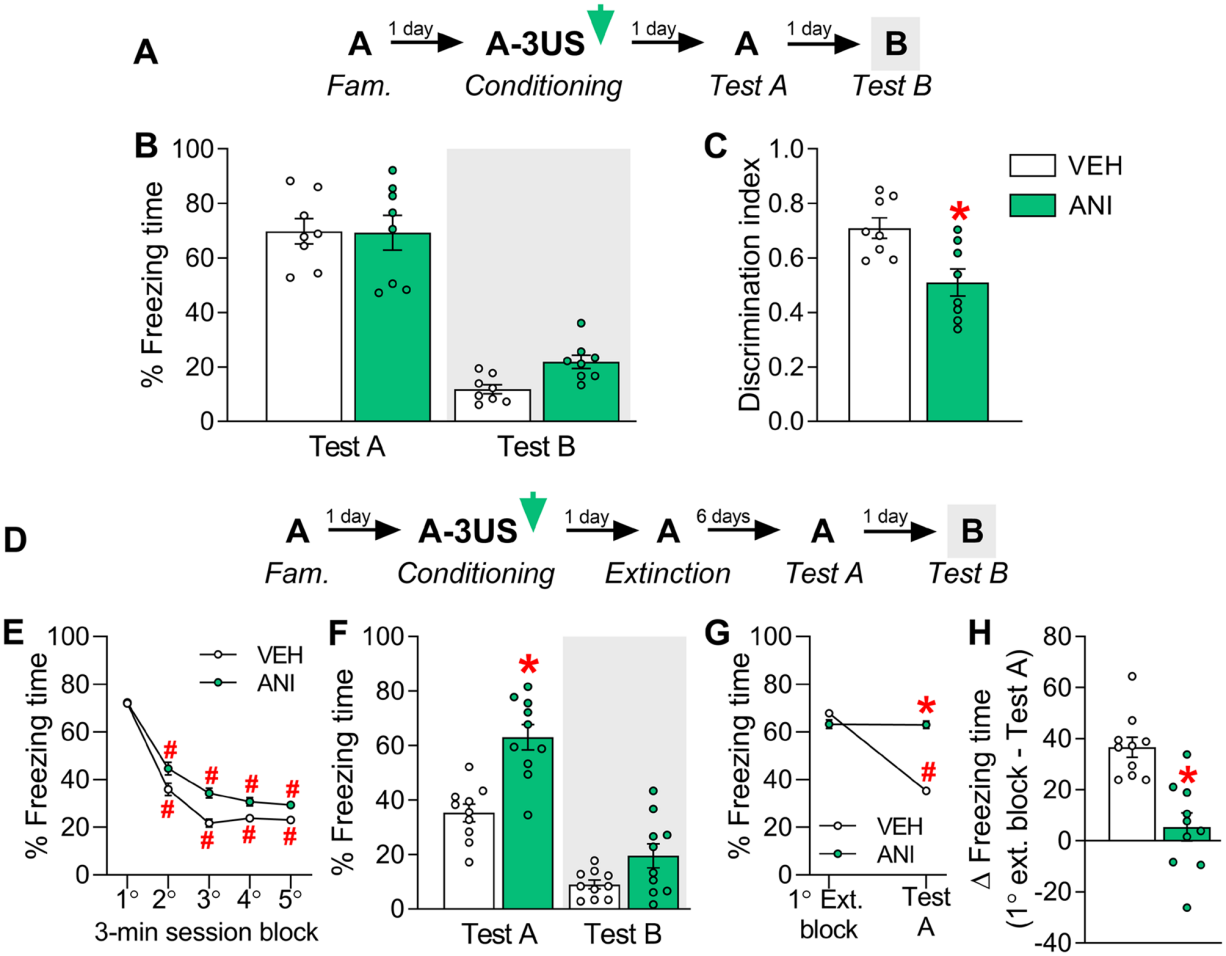
Effects of IL protein synthesis inhibition by anisomycin (ANI) during the consolidation of a contextual fear memory either on its expression in the paired context A (Test A) and the context B (Test B) (*upper graphs*) or on its extinction and Tests A and B (*lower graphs*). (**A**) Experiment’s 8 design. (**B**) ANI-treated animals presented comparable freezing times to controls (VEH) during Tests A and B (*p* = 0.14). (**C**) The ANI group presented lower discrimination index values than the VEH group. (**D**) Experiment’s 9 design. (**E**) The freezing time reduction throughout the extinction session in ANI-treated animals was similar to controls (VEH group). (**F**) ANI-treated animals did not retain the extinction learning as well as the VEH group, as observed during Test A. On Test B, no differences (*p* = 0.09) were found. (**G**) To estimate how efficient the extinction learning and memory process was, the time spent freezing in the first extinction session block and that expressed during Test A were compared. There was a difference indicative of a sufficient extinction in animals treated with VEH, but not ANI. As a result, the freezing time delta was lower in ANI-treated animals than controls (**H**). Values are expressed as individual units and/or mean ± S.E.M (n per group in experiment 8 = 8; and experiment 9 = 10). The # represents a significant difference (*p* < 0.05) from the respective first extinction session block (mixed ANOVA followed by the Newman–Keuls test). The * denotes a statistically significant difference from the respective control group (mixed ANOVA followed by the Newman–Keuls test or unpaired t-test).

To investigate whether IL's influence on memory consolidation depends on protein synthesis, 16 animals were randomly allocated to two groups based on the treatment (vehicle, n = 8; anisomycin, n = 8) given immediately after pairing the context A with three shocks (Fig. 6A). Tests A and B were performed on the following days.

A mixed ANOVA showed significant effects of test sessions (F_1,14_ = 177.3; *p* = 0.00001), but not treatment (F_1,14_ = 1.1; *p* = 0.31) or interaction between these factors (F_1,14_ = 1.8; *p* = 0.20), for freezing time. As shown in Fig. 6B, unprotected Newman–Keuls tests showed that anisomycin and vehicle groups presented similar values during both Tests A (*p* = 0.93; *g* = 0.03) and B (*p* = 0.14; *g* = 1.65). There were significant treatment effects for the discrimination index (*t*_1,14_ = 10.3; *p* = 0.006). As shown in Fig. 6C, drug-treated animals presented lower values than controls (*p* = 0.006*; g* = 1.60).

### Experiment 9: Effects of IL protein synthesis inhibition during the consolidation of a contextual fear memory on its subsequent extinction

To this aim, 20 animals were randomly allocated to two groups based on the treatment (vehicle, n = 10; anisomycin, n = 10) given immediately after pairing the context A with three shocks (Fig. 6D). On the next day, both groups underwent a 15-min extinction learning session. Tests A and B were performed six and seven days later, respectively.

For extinction session data, a mixed ANOVA showed significant effects of time-bin (F_4,72_ = 30.67; *p* = 0.000001), but not treatment (F_1,18_ = 1.36; *p* = 0.25) or interaction between these factors (F_4,72_ = 0.76; *p* = 0.55). As shown in Fig. 6E, both groups presented lower freezing time values from the second to the fifth extinction session block relative to their respective first one (*p* ≤ 0.0008; *g* ≥ 1.08). No differences between groups were found across any extinction session block (*p* ≥ 0.47; *g* ≤ 0.32).

For Tests A and B, a mixed ANOVA showed significant effects of test sessions (F_1,18_ = 120.2; *p* = 0.00001), treatment (F_1,18_ = 21.7; *p* = 0.0002), and their interaction (F_1,18_ = 7.4; *p* = 0.01). As shown in Fig. 6F, anisomycin-treated animals presented higher freezing time levels than controls during Test A (*p* = 0.0003; *g* = 2.20), but not Test B (*p* = 0.09; *g* = 0.99).

To estimate how efficient the extinction learning and memory process was, the time spent freezing in the first extinction session block (Fig. 6E) and that expressed during Test A (Fig. 6F) were compared. A mixed ANOVA showed significant effects of treatment (F_1,18_ = 6.6; *p* = 0.02), test sessions (F_1,18_ = 38.9; *p* = 0.00001), and their interaction (F_1,18_ = 21.5; *p* = 0.0002). As shown in Fig. 6G, there were reduced values in animals treated with vehicle (*p* = 0.0001; *g* = 3.37), but not anisomycin (*p* = 0.27; *g* = 0.36). Similarly, as shown in Fig. 6H, there were significant treatment effects on the freezing time delta (*t*_1,18_ = 21.5; *p* = 0.0002).

## Discussion

Inactivating the IL immediately after, but not six hours later, pairing the context A with either one or three shocks significantly reduced the ability to restrict freezing to the conditioned context, as seen during Test B performed 2 and 8 days after conditioning and inferred by the discrimination index. This result indicates that information processed in the IL, or at least passing through this medial prefrontal cortex subregion, during consolidation influences memory specificity. It is in line with that reported by Zelikowsky et al.^11^ and Xu and Südhof^12^, which lesioned the IL and inactivated both IL and PL, respectively, before contextual fear memory acquisition in rodents that later presented higher freezing times than controls when exposed to a novel and neutral context. Xu and Südhof^12^ have suggested a neural circuitry for fear memory specificity and generalization in which IL and PL modulate memory trace representations in the hippocampus through the thalamic nucleus reuniens. Based on this, the IL inactivation performed in our experiments has presumably affected the functional interaction of the neuronal circuitry underlying consolidation. Collectively, their data and ours suggest that the IL controls contextual fear memory specificity at acquisition and consolidation phases by suppressing generalization. Importantly, this view is consistent not only with animal and human cued fear conditioning results^36^ but also with the IL’s contribution to discriminate between fear and safety cues in both contextual and cued fear conditioning^37,38^. Of note, the post-acquisition IL inactivation-induced memory generalization was not associated with changes in anxiety-related behaviors. Besides, the IL and the adjacent PL typically have opposite roles in learning and memory processing^39^, a feature also valid to fear overgeneralization. Whereas stimulating the IL activity suppresses it^40^, a similar approach applied to the PL can promote it^41^.

Unlike Test B, there were no differences between muscimol and vehicle groups during Test A in any experiment conducted here (except experiment 7, in which it was carried out post-extinction). This result indicates that intensity is not a primary memory aspect influenced by the IL activity during consolidation. It is in line with that reported by Wang et al.^17^, which inactivated the IL with muscimol during the consolidation of inhibitory avoidance memory in animals that later behaved similarly to controls. In contrast, there was a relative reduction in inhibitory avoidance when the IL was inactivated with tetrodotoxin 25 min after training^16^. Differences in the duration of the pharmacological intervention used and the moment it was given may account for the mixed findings. Besides, the neural substrate regulating contextual fear conditioning and inhibitory avoidance is thought to be overlapping yet distinct^10^ and, thus, the relative IL contribution in each case may vary. Importantly, as previously mentioned, changes in IL plasticity patterns are associated with aversive memory consolidation. However, they do not necessarily influence memory intensity upon retrieval and expression. Instead, they could allow adjustments in mechanisms supporting the original aversive memory to retain the extinction one^21,42–44^. For instance, whereas fear conditioning was sufficient to depress the IL intrinsic excitability^18,19^, fear extinction produced the opposite outcome^45^. Of note, dampening the excitability of IL neurons is accompanied by impaired extinction consolidation^46^.

Animals in which the IL was inactivated during consolidation extinguished similarly to controls within the session but were unable to recall the extinction memory the following day. This result is in line with that found when the IL was lesioned or inactivated before cued and contextual fear memory acquisition^12–14^, suggesting that the IL controls the formation of extinction-sensitive fear memories at both stages. Of note, activity in the IL similarly has a role not only after consolidation, in maintaining extinction-sensitive fear memories^47^, but also during their extinction acquisition and consolidation^48–50^. Furthermore, post-extinction Test B data from muscimol-treated animals in experiment 7 and those from controls (without extinction) in experiments 1 and 5 are alike, suggesting that a single session of extinction learning was insufficient to attenuate the overgeneralization produced by IL inactivation after memory acquisition.

Our result showing that anisomycin-treated animals extinguished within the session but were unable to recall the extinction memory the following day is equivalent to that seen with muscimol and indicates that protein synthesis in the IL is necessary for consolidating extinction-sensitive contextual fear memories. This finding agrees with those relating de novo protein synthesis requirement in the IL to fear extinction memory consolidation^51,52^. Of note, reactivating the IL neuronal ensembles involved in the original memory engram during remote fear extinction is thought to be required for fear attenuation^53^. Similarly, recruiting the IL neuronal population activated during social interaction can alleviate conditioned fear responses^54^. Collectively, data suggest that activity and plasticity in the IL during initial learning stages are essential for posterior suppression of fear. In this context, future studies are guaranteed to examine remaining open questions. For instance, is the memory engram representing fear memory in the IL suppressed by another representing the extinction learning? Alternatively, is extinction learning achieved through changes in the original engram representing the fear memory?

Although there were no statistically significant differences in *p*-values between anisomycin and vehicle groups during Test A and Test B (the effect size was significant in this case) in experiment 8, a reduction in the discrimination index was shown. This result suggests that protein synthesis in the IL has moderate effects on specificity and generalization attributes of the memory during consolidation compared to those exerted on extinction retention. The study by Awad et al.^52^ used a similar approach (i.e., comparing lidocaine-induced temporary inactivation with anisomycin-induced protein synthesis inhibition) and found subtle quantitative differences as well. A possible explanation for the absence of anisomycin-induced effects on Test B is the dose of 20 μg/hemisphere used here. The dose range in which this drug produces significant effects when infused into the IL or PL varies from 12.5 to 62.5 μg/hemisphere^15,51,52,55,56^. However, that possibility is unlikely since the same dose interfered with extinction retention (experiment 9), being selected here because it provides more spatially precise protein synthesis inhibition than the dose of 62.5 μg/hemisphere^28^. Of note, we considered our results as if anisomycin primarily had impaired the protein synthesis in the IL. However, it is acknowledged that its administration can also interfere with other processes presumed to be necessary for cellular memory consolidation, including monoamine levels^57^ and neural activity^58^.

In summary, either suppressing the activity or inhibiting protein synthesis in the IL during contextual fear consolidation produces more generalized and extinction-resistant memories. At least three not mutually excluding potential explanations may account for this pattern of findings, namely: (i) it represents the direct IL influence over brain regions supporting aversive memory consolidation; (ii) it results from the lack of modulation of other interconnected areas of the underlying circuitry through the IL; and (iii) it is associated with a failure in establishing an inhibitory engram in the IL during memory consolidation. Whatever the case, the precise neural basis and plastic mechanisms underlying the IL-related changes reported here require further examination.

## Data Availability

All data supporting this study are available from the corresponding author upon request.
